# Difficultés de la prise en charge d’une thrombose veineuse cérébrale (TVC) compliquée d’hémorragie sous arachnoïdienne (HSA) chez le nourrisson: à propos d’un cas

**DOI:** 10.11604/pamj.2017.28.206.11639

**Published:** 2017-11-07

**Authors:** Idrissa Basse, Ndéye Rama Diagne/Gueye, Ndiémé Ndiaye, Marie Mbengue, Dina Cyrienne Obambi, Aïssatou Ba, Amadou Lamine Fall, Aliou Abdoulaye Ndongo, Djibril Boiro, Abou Ba

**Affiliations:** 1 Service de Pédiatrie, Hôpital pour Enfants de Diamniadio, Université de Thiès, Sénégal; 2 Service de Radiologie, Hôpital pour Enfants de Diamniadio, Sénégal; 3 Hôpital d’Enfants Albert Royer, Université Cheikh Anta Diop (CAD) de Dakar, Sénégal; 4 Service de Pédiatrie, Centre Hospitalier National Abass Ndao, Université CAD de Dakar, Sénégal

**Keywords:** Thrombose veineuse cérébrale, scanner, anticoagulant, enfant, Cerebral venous thrombosis, CT scan, anticoagulant, infant

## Abstract

La TVC est rare en général et particulièrement chez l’enfant. La survenue d’une HSA dans ce contexte est une situation très peu fréquente et seuls quelques cas sont rapportés dans la littérature. Sa symptomatologie est variable et souvent trompeuse. Non traitée ou en cas de traitement tardif l’issue peut être fatale ou conduire à des séquelles potentiellement graves. Nous rapportons l’observation d’un nourrisson de 22 mois reçu pour des convulsions avec coma stade II, syndrome d’hypertension intracrânienne et syndrome infectieux. Une septicémie à Pseudomonas spp a était retrouvée à la biologie et le scanner cérébral a permis de poser le diagnostic. Le traitement a été basé sur l’antibiothérapie mais surtout l’anticoagulation. Une nette amélioration clinique a été ainsi notée et le scanner cérébral de contrôle montrait une disparition de la thrombose avec images hémorragiques séquellaires droites. La TVC est une pathologie grave d’origine généralement infectieuse. Le traitement anticoagulant est aujourd’hui sujet à controverse notamment en cas d’hémorragie associée, mais l’expérience clinique serait en faveur de l’efficacité et de l’innocuité de ce traitement.

## Abstract (en)

CVT is generally rare, particularly among infants. The occurrence of a SAH associated with CVT is very uncommon and only a few cases have been reported in the literature. Symptoms are variable and often misleading. Outcome can be lethal or it can cause potentially severe sequelae if it is not treated or treated late. We report the case of a 22-month old infant examined for convulsions with Stage II coma, intracranial hypertension syndrome and infectious syndrome. Laboratory tests showed pseudomonas spp sepsis and cerebral CT scan allowed the diagnosis. Treatment was based on antibiotic therapy but mainly on anticoagulation therapy. The patient had a significant clinical improvement and follow-up cerebral CT scan showed disappearance of thrombosis with sequellar right hemoragic images. CVT is a severe disease, usually of infectious origin. Anticoagulant therapy is controversial, in particular in patients with associated hemorrhage, but clinical experience supports the effectiveness and safety of this treatment.

## Introduction

La TVC est rare en général et particulièrement chez l’enfant. Elle doit bénéficier d’un regain d’intérêt du fait de son polymorphisme clinique, de la multiplicité de ses étiologies et de sa gravité. Une publication d'une grande série canadienne a estimée l'incidence TVC chez les enfants à 0,67 pour 100 000 enfants avec 38% des enfants ayant des déficits neurologiques résiduels et une cause spécifique de mortalité de 4% [1]. Elle est d’une morbidité donc importante avec des séquelles neurologiques de l’ordre de 40% dans la série de Barnes 2004; 37,5% dans la série de Mariem 2007 [2, 3]. Le diagnostic a été amélioré ces dernières années grâce aux progrès de l’imagerie médicale constituée essentiellement par le scanner cérébral dans nos centres, l’IRM ou l’angiographie dans les plus grands centres spécialisés [4]. Le pronostic est intimement lié à une prise en charge rapide et adéquate basée sur le traitement étiologique, le traitement de l’hypertension intracrânienne mais surtout l’anticoagulation [5]. Les indications de cette anticoagulation restaient pendant longtemps sujet de controverse sont aujourd’hui mieux codifiées avec les consensus professionnels régulièrement actualisés basés sur les études observatoires, l’expérience des experts et l’analogie avec la pathologie d’adultes [6]. Nous rapportons cette observation de TVC survenue chez un nourrisson afin de faire le point sur le polymorphisme clinique, l’aspect étiologique, la place de l’imagerie précisément de la tomodensitométrie, la prise en charge et enfin le pronostic de cette affection.

## Patient et observation

**Figure 1:**
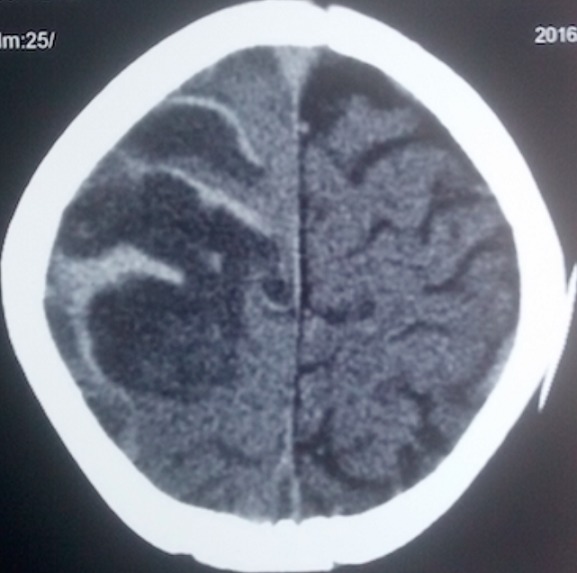
Scanner cérébral montrant l’hémorragie sous arachnoïdienne

**Figure 2:**
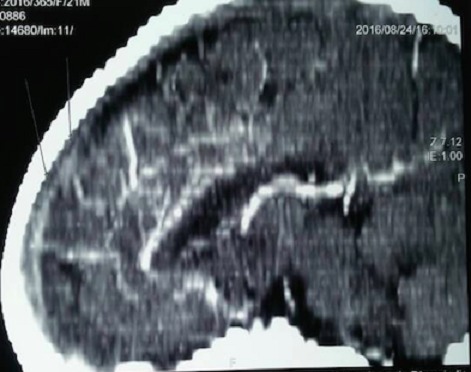
Scanner cérébral montrant la thrombose du sinus longitudinal

**Figure 3:**
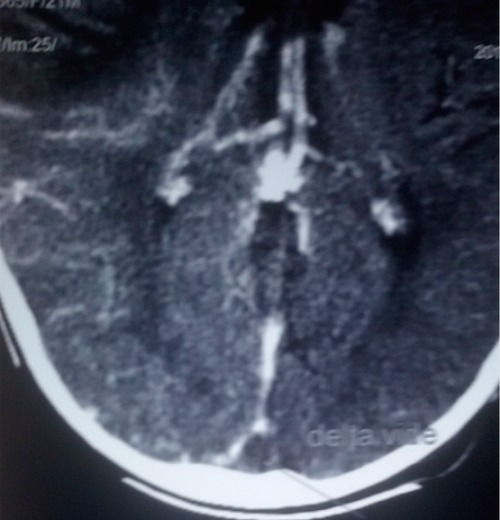
Scanner cérébral montrant le thrombus: signe du delta vide

**Figure 4:**
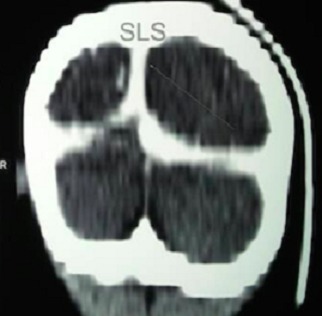
Scanner cérébral de contrôle montrant l’absence de thrombus au niveau du sinus longitudinal supérieur

Y.S.D. est un nourrisson de 22 mois de sexe masculin sans antécédent anténatal, périnatal et néonatal particulier avec un statut vaccinal à jour et un bon développement psychomoteur reçu pour des convulsions tonicocloniques généralisées associées à des vomissements. Son examen clinique notait un coma stade II avec un syndrome d’hypertension intracrânienne et une hémiparésie gauche; un syndrome de réponse inflammatoire systémique et un syndrome anémique. Le bilan biologique retrouvait un syndrome inflammatoire biologique avec une hyperleucocytose à polynucléaires neutrophiles et une CRP élevée, une anémie sévère hypochrome microcytaire avec un taux d’hémoglobine à 4,3 g/dL et une hyponatrémie légère. Le fond d’œil mit en évidence un œdème papillaire stade I. Ainsi, le scanner cérébral réalisé montra une hyperdensité spontanée des sillons corticaux fronto-pariétaux droits accompagnés d’une hypodensité du parenchyme sous cortical d’allure oedèmateuse (Figure 1), avec en plus un défaut de réhaussement du sinus longitudinal supérieur étendu au sinus latéral droit (Figure 2) et le signe du delta vide (Figure 3). Le diagnostic d’une thrombose veineuse cérébrale compliquée d’une hémorragie sous arachnoïdienne droite posé, nous réaliserons un bilan de la crase sanguine qui reviendra normal. Par ailleurs l’hémoculture qui a poussé au 4^ème^ jour de son hospitalisation isola un pseudomonas spp sensible aux béta lactamines. Ainsi un traitement fut institué avec une transfusion sanguine, le traitement anticonvulsivant fait de Diazépam et de phénobarbital, une antibiothérapie à base de Ceftriaxone et de gentamicine ainsi qu’une anticoagulation avec l’héparine de bas poids moléculaire et un antivitamine K. Du mannitol a également été administré pendant 3 jours pour lutter contre l’œdème cérébral. Son évolution a été marquée par un arrêt des convulsions, une reprise de la conscience, des muqueuses bien colorées avec cependant une persistance de la fièvre motivant une modification de l’antibiothérapie par l’association Ciprofloxacine et Amikacine. Son examen au 12^ème^ jour permettait de noter une apyrexie stable et une hémiparésie gauche que la kinésithérapie améliorait de jour en jour. Le scanner cérébral de contrôle fait après 12 semaines d’anticoagulation montrait une disparition de la thrombose (Figure 4) avec images de séquelles hémorragiques à droite.

## Discussion

La thrombose veineuse cérébrale a été décrite par Ribes pour la première fois en 1825 [7]. Pendant de longues années, elle a été considérée comme étant une maladie infectieuse qui entraînait une occlusion du sinus sagittal supérieur ou sinus longitudinal supérieur. C’est tout d’abord la révolution des procédures d’imagerie qui a essentiellement permis le diagnostic fiable de TVC et qui a contribué pour beaucoup à la compréhension du tableau clinique [8]. Dans la population générale, l’incidence de la TVC est évaluée à environ 1-2/100000 d’après l’étude multicentrique, prospective, la plus importante à ce jour (International Study on Cerebral Vein and Dural Sinus Thrombosis, ISCVT) [9]. Dans des centres tertiaires, trois à huit cas sont traités annuellement [10]. Chez les enfants l’incidence estimée à partir du plus grand registre pédiatrique est de 0,67 pour 100000. Cependant une incidence plus élevée chez les nouveau-nés a été rapportée par différentes études (2,6-12 pour 100 000 nouveau-nés par an) [11,12].

En Afrique en dehors de l’étude de DAMAK M. qui a rapporté une série de 16 cas en 14 ans très peu de cas ont été décrits. Dans le travail de Mahendranath M. chez le nouveau né on notait une prédominance masculine avec 72% des cas [1]. Du point de vue physiopathologique les manifestations de la TVC relèvent de deux mécanismes qui se produisent simultanément dans la majorité des cas. Le premier mécanisme implique l'occlusion des veines cérébrales, qui provoque l'œdème localisé du cerveau et l'infarctus veineux. Dans le deuxième mécanisme, l'hypertension intracrânienne se développe à la suite de l'occlusion des principaux sinus veineux [13]. Les symptômes d’hypertension intracrânienne, les signes neurologiques et l’œdème papillaire retrouvés chez notre malade ont été décrits dans la majorité des cas de TVC [3, 4]. Le scanner cérébral, examen de 1^ère^ intention, a montré le signe du triangle dense (Figure 4). Le réseau veineux concerné (sinus longitudinal supérieur et sinus latéral) se retrouve dans plus de 70% des cas. L’hémorragie sous arachnoïdienne associée, décrite comme étant une conséquence de la stase veineuse est peu fréquente [2]. L’IRM cérébrale constitue l’examen de référence mais est d’accès limité dans notre pays. La TVC est une affection multifactorielle, les étiologies sont multiples dominées chez l’enfant par les troubles prothrombotiques héréditaires ou acquis ainsi que les hémolyses chroniques comme la thalassémie ou la drépanocytose. Dans ce cadre une électrophorèse de l’hémoglobine a été faite chez notre patient montrant un profil normal. La fonction hépatique était également normale de même que les plaquettes. Ainsi, avec le contexte de septicémie l’étiologie infectieuse semblait plus probable bien qu’un bilan plus exhaustif soit nécessaire dans le cadre du suivi. En effet avec l’avènement des antibiotiques les causes infectieuses sont de plus en plus rares dans les pays occidentaux cependant elles prédominent en Afrique comme le prouvent les études de Damak et Khabbache en Tunisie [3,14]. Les infections initialement « bénignes » du scalp cérébral, ORL ou méningées, pouvant se compliquer de TVC, doivent bénéficier d’une PEC précoce et efficace surtout dans nos pays en développement où les facteurs de risque d’infections restent importants.

Le traitement repose sur l’anticoagulation dont l’utilisation a été longtemps controversée mais selon les données actualisées elle reste recommandée surtout à la phase aiguë des TVC de l’enfant même en cas de complication hémorragique associée. L’anticoagulation est probablement efficace pour réduire le risque de décès et de séquelles (classe IIa, niveau d’évidence B). Pour le choix de l’anticoagulant il ressort de la littérature que chez le jeune enfant et le nourrisson, l’utilisation d’héparines de bas poids moléculaire est plus facile, y compris au long court [6]. Ces recommandations sont en phase avec ce que nous avons rapporté dans notre observation où l’anticoagulation poursuivie pendant 6 mois a permis d’avoir une récupération plus ou moins complète des fonctions neurologiques. Cependant le traitement associé garde une importance capitale, il comporte la correction de l’anémie, la lutte contre l’œdème cérébral mais surtout une antibiothérapie qui doit être adaptée à l’antibiogramme mais également à l’écologie bactérienne locale. Le pronostic est marqué par une morbidité résiduelle avec présence de déficits neurologiques dans près de 40% des cas [3,11] et une mortalité de 4 à 33% [4]. Notre difficulté en pratique reste le diagnostic précoce avant l’apparition de lésions parenchymateuses afin de réduire le risque de complications et de préserver le pronostic vital et fonctionnel.

## Conclusion

Dans notre centre pédiatrique de niveau 3, en 4 ans cette observation représente le premier cas de thrombose veineuse cérébrale diagnostiquée. Donc il s’agit d’une affection peu fréquente avec une présentation clinique non spécifique et très variable qui rend difficile son diagnostic. Le scanner cérébral sans et avec injection de produit de contraste est d’un apport considérable, d’autant plus que les examens de choix que sont l’imagerie par résonnance magnétique et l’angiographie nous sont peu accessibles. Aucune étiologie n’est retrouvée dans prés de 15% des cas de TVC mais dans nos populations les causes infectieuses restent prédominantes. Le traitement symptomatique demeure plus ou moins uniforme dans la majorité des études mais l’anticoagulation revêt quelques zones d’ombre notamment dans les formes avec hémorragie associée ou chez le nouveau né. Cependant le bénéfice de l’héparinothérapie est bien démontré et le pronostic est bon si le diagnostic est fait précocement et le traitement administré à temps.

## Conflits d’intérêts

Les auteurs ne déclarent aucun conflit d’intérêts.
